# Multiple Sclerosis Identification Based on Fractional Fourier Entropy and a Modified Jaya Algorithm

**DOI:** 10.3390/e20040254

**Published:** 2018-04-05

**Authors:** Shui-Hua Wang, Hong Cheng, Preetha Phillips, Yu-Dong Zhang

**Affiliations:** 1 School of Computer Science and Technology, Henan Polytechnic University, Jiaozuo 454000, China; 2 Department of Informatics, University of Leicester, Leicester LE1 7RH, UK; 3 Department of Electrical Engineering, the City College of New York, CUNY, New York, NY 10031, USA; 4 Department of Neurology, First Affiliated Hospital of Nanjing Medical University, Nanjing 210029, China; 5 West Virginia School of Osteopathic Medicine, 400 N Lee St, Lewisburg, WV 24901, USA

**Keywords:** multiple sclerosis, Jaya algorithm, cost-sensitive learning, fractional Fourier entropy, multilayer perceptron, feedforward neural network, *k*-fold cross validation

## Abstract

*Aim*: Currently, identifying multiple sclerosis (MS) by human experts may come across the problem of “normal-appearing white matter”, which causes a low sensitivity. *Methods*: In this study, we presented a computer vision based approached to identify MS in an automatic way. This proposed method first extracted the fractional Fourier entropy map from a specified brain image. Afterwards, it sent the features to a multilayer perceptron trained by a proposed improved parameter-free Jaya algorithm. We used cost-sensitivity learning to handle the imbalanced data problem. *Results*: The 10 × 10-fold cross validation showed our method yielded a sensitivity of 97.40 ± 0.60%, a specificity of 97.39 ± 0.65%, and an accuracy of 97.39 ± 0.59%. *Conclusions*: We validated by experiments that the proposed improved Jaya performs better than plain Jaya algorithm and other latest bioinspired algorithms in terms of classification performance and training speed. In addition, our method is superior to four state-of-the-art MS identification approaches.

## 1. Introduction

Multiple sclerosis (MS) is a lifelong condition that always affects the brain and sometimes, but not always, the spinal cord [1]. It may cause various potential symptoms, including visual problems [2], spasms [3], numbness [4], fatigue [5], etc. MS is typically diagnosed by the presenting symptoms, together with supporting neuroimaging methods, such as magnetic resonance imaging (MRI) to detect the damaged white matter (WM).

Nevertheless, MS diagnosis is difficult, since it may be confused with other white matter diseases, such as neuromyelitis optica (NMO), acute disseminated encephalomyelitis (ADEM), acute cerebral infarction (ACI), etc. For example, the spinal cord lesions in MS patients are typically oval, peripheral, and asymmetric, but in NMO patients, they are longitudinally extensive and centrally located. The mean number of involved vertebral segments in NMO patients is significantly more than that in MS patients. Furthermore, the number of spinal cord lesions in MS patients is remarkably more than in NMO.

In this study, we carried out a preliminary study that differentiates MS with healthy controls. It is a physically and mentally laborious task for neuro-radiologists to identify MS from healthy brains. As is known, computers perform better than human in terms of machine vision, since the computer machines can identify slight brightness change, and perceive slight structural change [6,7]. In the last decade, scholars have proposed many computer-vision based methods to identify MS from healthy brains.

For instances, Ghribi et al. [8] proposed a segmentation method based on gray-level co-occurrence matrix (GLCM) and gray-level run length (GLRL) methods. Murray et al. [9] presented a multiscale amplitude-modulation frequency-modulation (MAMSM) method. Nayak et al. [10] offered a random forest (RF) approach. Lopez and co-workers [11] employed Haar wavelet transform (HWT) and logistic regression (LR). They found three-level decomposition performed the best.

To identify MS in a more efficient and accurate way, we consider to use a relatively new feature extraction method—fractional Fourier entropy (FRFE) [12], which combines the fractional Fourier transform (FRFT) and Shannon entropy. The reason why we used FRFE is due to its super-effectiveness for fine-grained classification. Besides, we proposed an idea of FRFE map based on different combination angles. Multilayer perceptron (MLP) was chosen as the classifier because of the universal approximation theory, which states that MLP can approximate to any function in any degree.

In addition, we proposed a modified Jaya algorithm to further train the MLP. Jaya was chosen since it does not need to set the algorithm-specific parameters. In this paper, we proposed two improvements, so this improved Jaya algorithm does not need to set population size and number of hidden neurons.

The following contents are structured as follows: Section 2 gives the two brain imaging datasets, describes the subjects used, and introduces the inter-scan normalization and cost-sensitive learning. Section 3 presents the methodology, including the FRFE spectrum map and our proposed Self-adaptive Three-segment-encoding Jaya algorithm. Section 4 covers the experiments and results. Finally, Section 5 is devoted to conclusion and future directions.

## 2. Materials

### 2.1. Source from Internet

**Figure 1:**
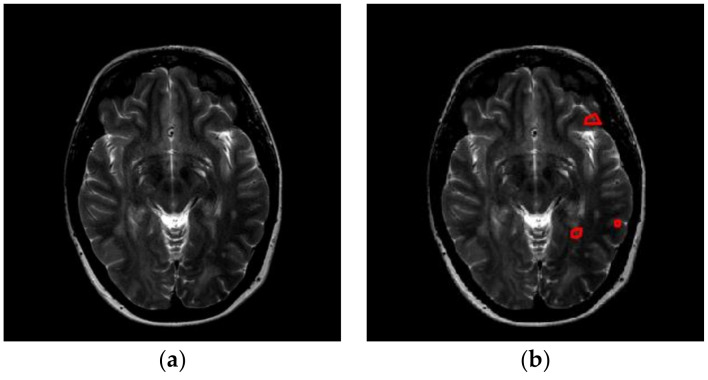
A slice with three plaques (areas surrounded by red lines denote the plaque). (**a**) Original Image; (**b**) Delineated.

**Figure 2:**
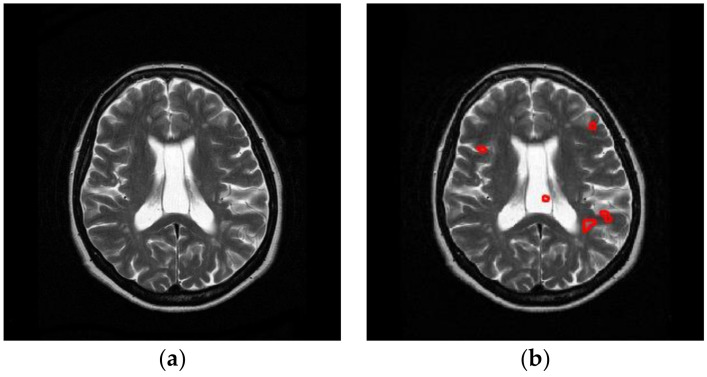
A slice with five plaques (areas surrounded by red lines denote the plaque). (**a**) Original Image; (**b**) Delineated.

The images used in this study come from two sources. First, we downloaded brain images from the eHealth laboratory [13]. There are 38 patients (aged 34.1 ± 10.5 years, 17 males and 21 females) in the dataset. All brain lesions were identified by experienced MS neurologists, and were confirmed by radiologists. We selected the slices that were associated with plaques, and finally obtained 676 slices altogether. Figure 1 and Figure 2 shows two slices with three and five plaques, respectively. The expanded disability status scale (EDSS) of starting scores are 2.2 ± 0.8. After five years, the EDSS scores of all 38 patients were 2.85 ± 1.5 [14].

### 2.2. Source from Local Hospitals

The above dataset did not contain healthy controls. Hence, we enrolled healthy controls (HC) who fell in the same range of age and gender distribution of the first dataset. It is difficult to identify and enroll MS patients for MRI scans, but healthy controls are available more easily, and they are compliant during MRI scan. In the computer server of our hospital, we have imaging data of thousands of healthy controls. The exclusion criteria for all volunteers were known neurological or psychiatric diseases, brain lesions, taking psychotropic medications, and contraindications to MR imaging. Our study was approved by the Ethics Committee of the participating hospitals, and a signed informed consent form was obtained from every subject prior to entering this study. Then, we selected 880 slices from 34 HCs (aged 33.6 ± 9.7 years, 16 males and 18 females).

We did not increase the size of the healthy subject database, because the 880 slices are sufficient compared to the 676 slices of MS patient cohort. If we collect more healthy subjects, the whole dataset will be severely imbalanced, and making following classifier model difficult to establish.

### 2.3. Inter-Scan Normalization and Cost-Sensitive Learning

As we have two sources of brain imaging data, the scanner machines may have different hardware and/or software parameters, we need to match the two sources of images in terms of gray-level intensities. In this study, we used the histogram stretching (abbreviated as HS) [15] method. Suppose *d* is the original brain image, and *e* is the normalized image, we have:

(1) $$e(x,y)=\frac{d(x,y)-d_{\min}}{d_{\max}-d_{\min}}$$

where (*x*, *y*) the coordinate of pixel, *d*_min_ and *d*_max_ represents the minimum and maximum intensity values of original image *d*. Finally, we have a dataset of 676 + 880 = 1556 brain images. 

Since the number of images of two classes are different, we used cost matrix [16] to balance the effect of this imbalanced dataset. The cost of HC was set to 1, and the cost of MS was set to 880/676 = 1.30, as shown in Table 1.

## 3. Methodology

### 3.1. Fractional Fourier Entropy

The fractional Fourier entropy (FRFE) is a relatively new feature extraction method. It combines both the fractional Fourier transform (FRFT) and Shannon entropy. It has successfully been applied to tea category identification [17], disease detection [18], hearing loss identification [19].

Take one-dimensional signal as an example, assume *v*(*t*) is a one-dimensional signal, *t* is the time domain, and *q* is the frequency domain. We have the FRFT defined as:

(2) $$Q_{a}(q)=\int_{-\infty }^{\infty }v(t)N(t,q|a)dt$$

here *Q* represents the FRFT result, and *a* the angle of FRFT. N is the transform kernel as:

(3) $$N(t,q|a)=\sqrt{1-ℐ\cot a}\times \exp\left[ℐ\pi \times (t^{2}\times \cot a-2q\times t\times \csc a+q^{2}\times \cot a)\right]$$

Here ℐ represents the imaginary unit, and exp(.) represents the exponential function. 

As is familiar to readers that if *a* is set the value of a multiple of π, then both “csc” and “cot” operators will diverge to infinity. We can transform Equation (3) via this knowledge as:

(4) $$N(t,q|a)=\{\begin{array}{c} ℋ(t-q)\\ \sqrt{1-ℐ\cot a}\times \exp\left(ℐ\pi (t^{2}\cot a-2qt\csc a+q^{2}\cot a)\right)\\ ℋ(t+q) \end{array}\;\mathrm{if}\;\frac{a}{\pi }\{\begin{array}{c} =2c\\ \neq c\\ =(2c+1)\pi \end{array}$$

where ℋ represents the Dirac delta function, and *c* represents an arbitrary integer.

The entropy was then performed over the fractional spectrum *Q*, and we obtained the final FRFE result as:

(5) $$F=\underset{q}{Entropy}\left[Q_{a}(q)\right]$$

where *F* is the FRFE measure.

### 3.2. FRFE Map

For one-dimensional signal, the angle value is scalar. For example, suppose we have a rectangular function *o*(*t*) defined as:

(6) $$o(t)=\{\begin{array}{c} 0\\ 1\\ 1/2 \end{array},\;\mathrm{if}\;|t|\left\{\begin{array}{c} >\\ <\\ = \end{array}\right\}\frac{1}{2}$$

**Figure 3:**
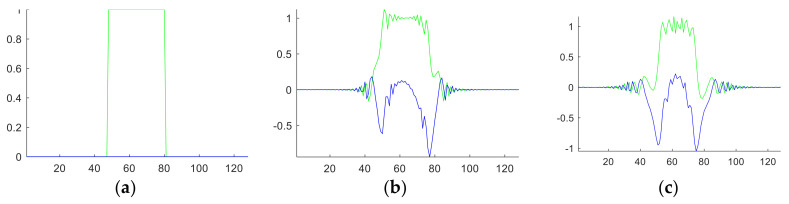

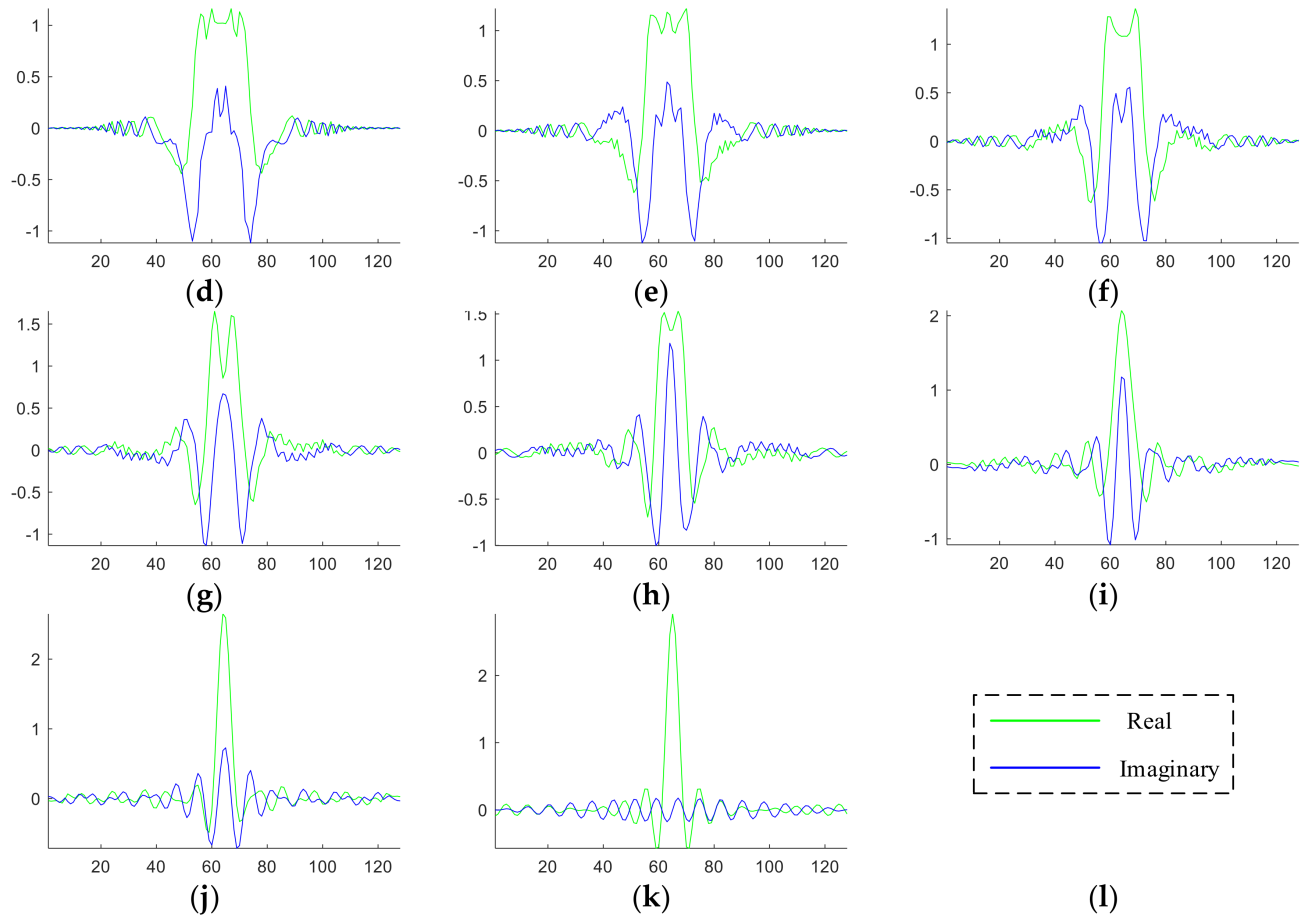
FRFT results of a rectangular function. (**a**) *a* = 0; (**b**) *a* = 0.1; (**c**) *a* = 0.2; (**d**) *a* = 0.3; (**e**) *a* = 0.4; (**f**) *a* = 0.5; (**g**) *a* = 0.6; (**h**) *a* = 0.7; (**i**) *a* = 0.8; (**j**) *a* = 0.9; (**k**) *a* = 1.0; (**l**) legend. The *x*-axis represents the time domain, and the *y*-axis represents the signal amplitude.

The FRFT results *Q_a_*(*q*) with *a* of from 0 to 1 with increase of 0.1 are shown in Figure 3. In this figure, the green line represents the real part, and the blue line represents the imaginary part of the FRFT results.

**Figure 4:**
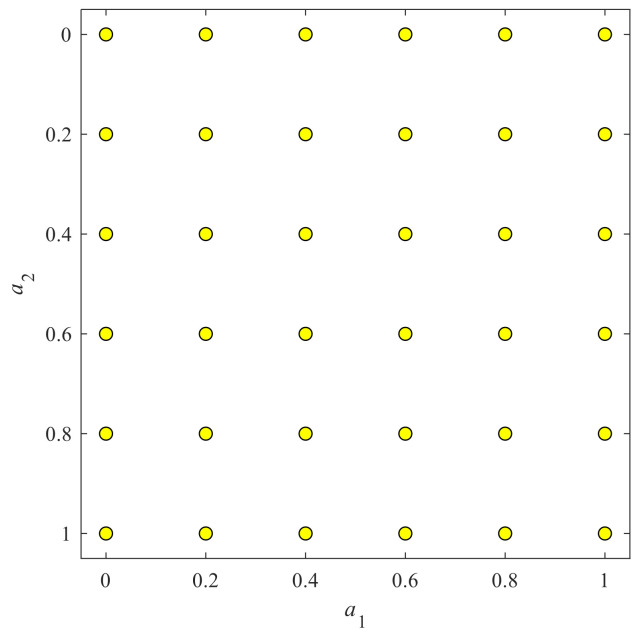
Vector-angle locations in a 2D map.

When the signal comes to the 2D situation, the angle *a* is now a two-element vector *a* = (*a*_1_, *a*_2_). This angle vector served as rotation angle for a 2D image when performing 2D-FRFT. To balance the computation complexity and classification accuracy, we finally selected a grid map as shown in Figure 4. Here we chose 36 vector-angles. For each dimension, the value varies from 0 to 1 with increase of 0.2, and hence we have six values for one dimension. After combination, we have altogether 36 vector-angles, viz., (0, 0), (0, 0.2), …, (0, 1), (0.2, 0), (0.2, 0.2), …, (0.2, 1), …, (1, 0), (1, 0.2), …, (1, 1). The pseudo-codes of 2D-FRFE were presented in Algorithm 1.
**Algorithm 1.** Pseudocode of 2D FRFE
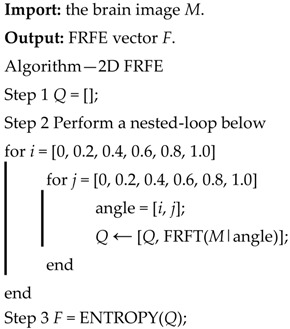



### 3.3. Multilayer Perceptron

A multilayer perceptron (MLP) is a type of two-layer feedforward neural network mapping input training to target labels [20]. The accuracy of MLP can be guaranteed by universal approximation theorem. MLP is a kind of shallow network. The reason why we do not use deep neural network is Occam’s razor [21]. Another reason is our dataset is comparatively smaller than those million-image datasets used in deep learning. The small number of images may impair the convergence of deep neural network.

**Figure 5:**
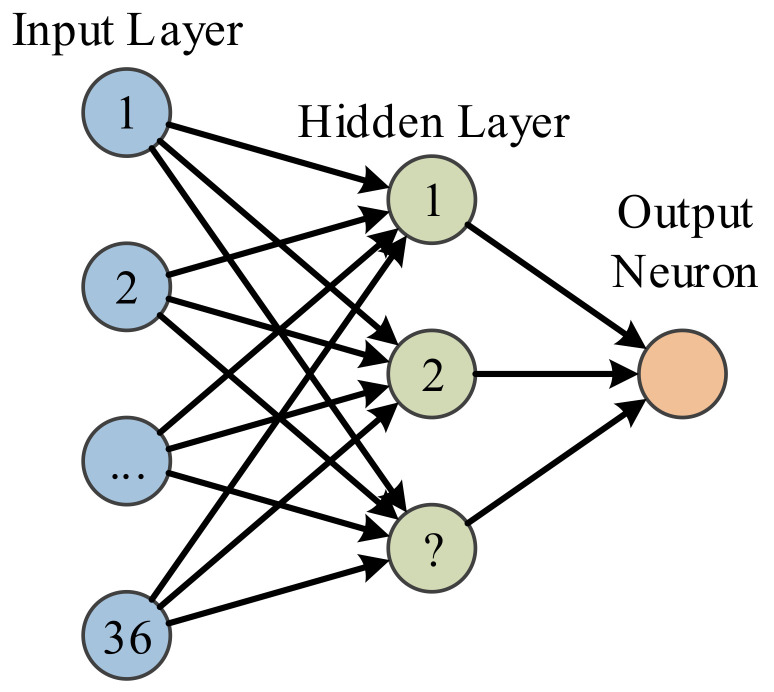
Structure of MLP.

MLP with structure shown in Figure 5 just shows this is a two-layer feedforward neural network, the output neuron is not a layer since it is not associated with weights. The input layer has 36 neurons linked to the 36 features extracted by FRFE. The number of hidden layer is a parameter to be optimized. The one output neuron shall output value either 1 or 0, indicating MS or healthy brain.

### 3.4. Jaya Algorithm

Current training methods of MLP include back propagation gradient descent and its variants. The gradient descent may be trapped into local minimum and saddle points. Hence, bioinspired methods were developed to train MLP, such as genetic algorithm [22], particle swarm optimization (PSO) [23], dynamic PSO (dPSO) [24], and biogeography-based optimization [25]. Nevertheless, those algorithms suffer from a main problem: how to set the hyperparameters of the algorithms themselves?

**Figure 6:**
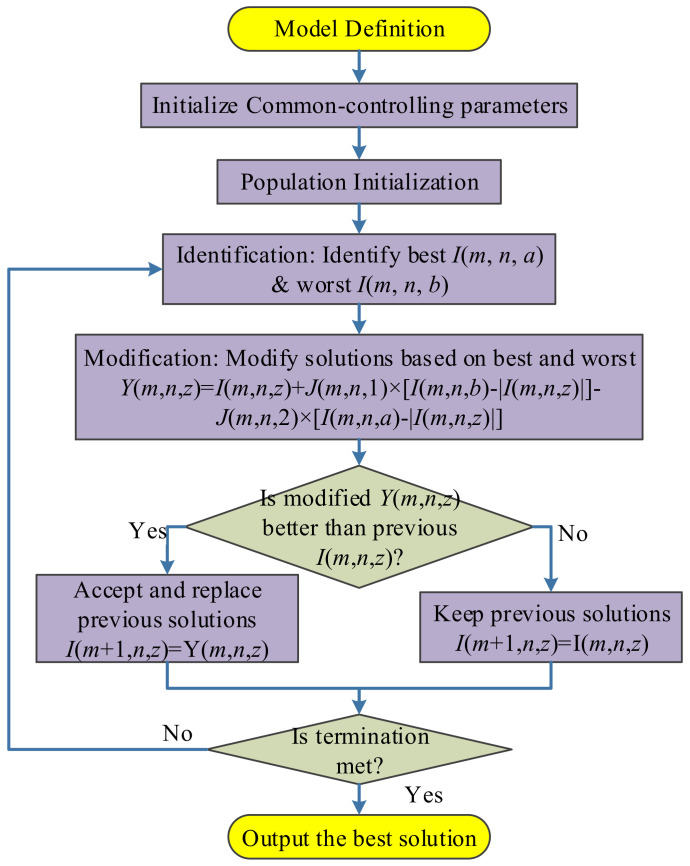
Flowchart of Jaya.

Jaya algorithm is a new optimization method proposed by Rao [26]. It divides all the hyperparameters into two types: algorithm specific parameter (ACP) and common controlling parameter (CCP). The success of Jaya lies in it only needs to set the values of CCP (the size of population and, the maximum iteration), and does not need to set the values of ASP [27]. The flowchart of Jaya was depicted in Figure 6.

Let us suppose *m*, *n*, *z* be the index of iteration, variable, and candidate. Suppose *J*(*m*, *n*, 1) and *J*(*m*, *n*, 2) are two random positive numbers in the range of [0, 1]. Assume *I*(*m*, *n*, *z*) represents the *n*-th variable of *z*-th solution candidate at *m*-th step. Suppose *a* and *b* denotes the index of worst and best candidate within the population, respectively:

(7) $$b=\underset{z}{\arg\min}\left[I(m,n,z)\right]$$

(8) $$a=\underset{z}{\arg\max}\left[I(m,n,z)\right]$$

Hence, *I*(*m*, *n*, *a*) and *I*(*m*, *n*, *b*) denotes the worst and best value of *n*-th variable at *m*-th iteration.

We can define the modified solution at each step *Y*(*m*, *n*, *z*) as:

(9) $$\begin{array}{l} Y(m,n,z)=I(m,n,z)+\\ J(m,n,1)\times \left[I(m,n,b)-\left|I(m,n,z)\right|\right]-\\ J(m,n,2)\times \left[I(m,n,a)-\left|I(m,n,z)\right|\right] \end{array}$$

**Figure 7:**
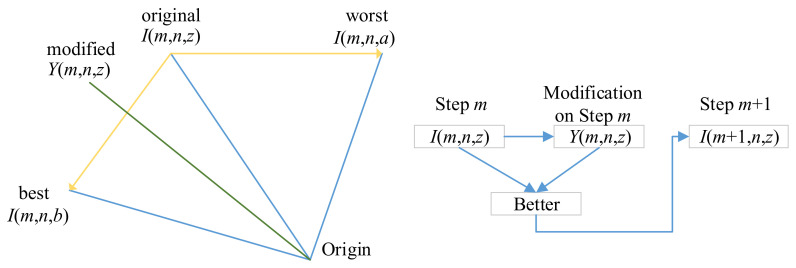
A toy example of solution update.

The 2nd term “*J*(*m*, *n*, 1) *×* [*I*(*m*, *n*, *b*) − |*I*(*m*, *n*, *z*)|]” in Equation (9) represents that the candidate needs to move closer to the best one. In contrast, the 3rd term “−*J*(*m*, *n*, 2) *×* [*I*(*m*, *n*, *a*) − |*I*(*m*, *n*, *z*)|]” in Equation (9) represents that the candidate needs to move away from the worst candidate, noting the “−” symbol before *J*(*m*, *n*, 2). Figure 7 shows an example that the best solution *I*(*m*, *n*, *b*) will push *I*(*m*, *n*, *z*) towards down-left direction, and the worst solution *I*(*m*, *n*, *a*) pushes towards left. 

In a word, the algorithm tries to get closer to success, *I*(*m*, *n*, *b*), and avoid failure, *I*(*m*, *n*, *a*). Hence, the algorithm attempts to select the best solution, and hence it is dubbed as Jaya (A Sanskrit word meaning victory). Rao [26] tested Jaya algorithm on 24 constrained benchmark functions in the Congress on Evolutionary Computation (CEC 2006). The comparison algorithms include homomorphous mapping, genetic algorithm, differential evolution, artificial bee colony, biogeography-based optimization, multi-membered evolution strategy, particle swarm optimization, and adaptive segregational constraint handling evolutionary algorithm. Their results showed Jaya secured first rank for the “best” and “mean” solutions in Friedman’s rank test for all 24 constrained benchmark problems.

The updated candidate at iteration (*m* + 1) can be written as:

(10) $$I\left(m+1,n,z\right)=\{\begin{array}{c} Y(m,n,z)\\ I(m,n,z) \end{array},\;\mathrm{if}\;\{\begin{array}{c} G\left[Y(m,n,z)\right]<G\left[I(m,n,z)\right]\\ G\left[Y(m,n,z)\right]\geq G\left[I(m,n,z)\right] \end{array}$$

where G represents the fitness function.

Equation (10) indicates that *I*(*m* + 1, *n*, *z*) is assigned with *Y*(*m*, *n*, *z*) if the modified candidate *Y*(*m*, *n*, *z*) is better in terms of fitness than *I*(*m*, *n*, *z*), otherwise it is assigned with *I*(*m*, *n*, *z*) [28]. The Jaya algorithm loops until the termination criterion is met. We set the termination criterion to either the algorithm reaches maximum iteration epoch, or the error does not reduce for five epochs.

### 3.5. Two Improvements

To further improve the performance of Jaya, and to fit to our problem, we presented an improved Jaya algorithm based on two points: (i) We use self-adaptive to automatic determine the size of population, and thus we only decide the maximum iteration number; (ii) We embed the three-segment-encoding strategy to optimize both weights, biases, and the number of hidden neurons in the MLP.

In the first improvement, suppose the number of design variable is *l*, the self-adaptive mechanism [29] initialize the population size *S* as:

(11) $$S_{0}=10\times l$$

Afterwards, the population size is dynamically adjusted following the formula

(12) $$S_{m+1}=\mathrm{round}\left(S_{m}+r\times S_{m}\right)$$

where *r* is random variable with value between [−0.5, 0.5].

Now the population size is automatically determined without user intervention. If the new population size is larger than older one (*S_m_*_+1_ > *S_m_*), then all the existing population will go to the next population, and the optimal solution in current population are assigned to the remaining (*S_m_*_+1_ − *S_m_*) solutions. If the size of new population is smaller than older one (*S_m_*_+1_ < *S_m_*), then only the best *S_m_*_+1_ solutions are transferred to the next population. No changes will happen if current population size is equal to next population size (*S_m_*_+1_ = *S_m_*).

In extreme conditions, if the number of next population decreases even less than the number of design variables (*l*), we need to increase it to *l*, viz., if *S_m_*_+1_ < *l*, then *S_m_*_+1_ = *l*. The term self-adaptive refers to the automatic selection of the population size.

**Figure 8:**
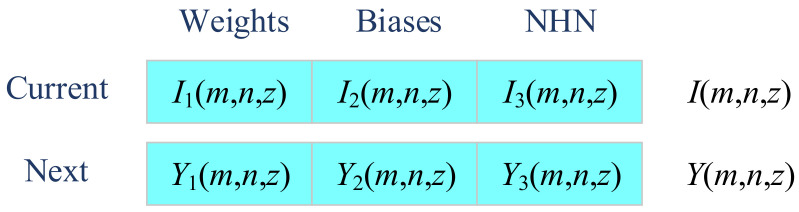
Illustration of three-segment encoding.

The second improvement is to embed a three-segment-encoding strategy [30], which optimizes the weights (Segment 1), biases (Segment 2), and number of hidden neurons (Segment 3) simultaneously. Using this method, solution *I*(*m*, *n*, *z*) is now comprised of three segments as:

(13) $$I(m,n,z)=\left[\begin{array}{ccc} I_{1}(m,n,z) & I_{2}(m,n,z) & I_{3}(m,n,z) \end{array}\right]$$

where *I*_1_(), *I*_2_(), and *I*_3_() represents extract the first part, second part, and third part of the solution candidate representation. *I*_1_(*m*, *n*, *z*) encodes the weights, *I*_2_(*m*, *n*, *z*) encodes the biases, and *I*_3_(*m*, *n*, *z*) encodes the number of hidden neurons (NHN). Similarly, the modified solution is defined as:

(14) $$Y(m,n,z)=\left[\begin{array}{ccc} Y_{1}(m,n,z) & Y_{2}(m,n,z) & Y_{3}(m,n,z) \end{array}\right]$$

where *Y*_1_(), *Y*_2_(), and *Y*_3_() encodes the weights, biases, and NHN of next iteration. Figure 8 shows the illustration of three-segment encoding.

The modification rule does not follow Equation (9). The new modification rule is three-folds as described in following three equations. A caveat is *K* and *T* are two random positive numbers, similar to variable *J*:

(15) $$\begin{array}{l} Y_{1}(m,n,z)=I_{1}(m,n,z)+J(m,n,1)\times \left[I_{1}(m,n,b)-\left|I_{1}(m,n,z)\right|\right]-\\ J(m,n,2)\times \left[I_{1}(m,n,a)-\left|I_{1}(m,n,z)\right|\right] \end{array}$$

(16) $$\begin{array}{l} Y_{2}(m,n,z)=I_{2}(m,n,z)+K(m,n,1)\times \left[I_{2}(m,n,b)-\left|I_{2}(m,n,z)\right|\right]-\\ S(m,n,2)\times \left[I_{2}(m,n,a)-\left|I_{2}(m,n,z)\right|\right] \end{array}$$

(17) $$\begin{array}{l} Y_{3}(m,n,z)=I_{3}(m,n,,z)+L(m,n,,1)\times \left[I_{3}(m,n,,b)-\left|I_{3}(m,n,,z)\right|\right]-\\ T(m,n,,2)\times \left[I_{3}(m,n,,a)-\left|I_{3}(m,n,,z)\right|\right] \end{array}$$

Considering both improvements, we name our method as Self-adaptive Three-segment-encoded Jaya (ST-Jaya). In the experiments, we shall compare this proposed ST-Jaya with state-of-the-art approaches.

### 3.6. Implementation

**Figure 9:**
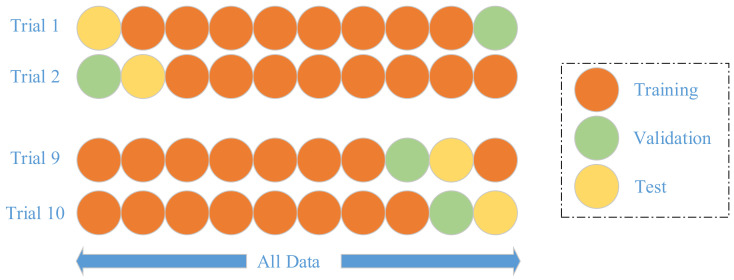
Illustration of 10-fold cross validation.

We do not segment the whole dataset into training and test set, since our dataset is already quite small. Instead, we employ a *k*-fold cross validation method [31], where *k* equals to 10 following conventions. *k*-fold cross validation is a strict model validation approach available in statistics textbooks. In *k*-th trial, the (*k* − 1)-th fold is used as validation, *k*-th fold as test, other folds are used as training set, as shown in Figure 9. Hence, the training, validation, and test folds in each trial are always independent.

The training iterates until the accuracy over validation set increases for five continuous epochs. Hence, the training set is used for learning the weights, biases, and NHN. The validation set is used for learning the maximum iteration number. The test set is used for reporting unbiased error.

### 3.7. Evaluation

Finally, our method was implemented in this 10-fold cross validation and cost-sensitivity learning way. Suppose *f* represents the number of folds, *t* the time of runs. We can deduce the ideal confusion matrix *E* of one time 10-fold cross validation:

(18) $$E(f=10,t=1)=\left[\begin{array}{cc} 676 & 0\\ 0 & 880 \end{array}\right]$$

We repeated it ten times and performed a 10 × 10-fold cross validation in realistic. Therefore, the confusion matrix is:

(19) $$E(f=10,t=10)=\left[\begin{array}{cc} 6760 & 0\\ 0 & 8800 \end{array}\right]$$

Suppose the positive class is multiple sclerosis (MS), and the false class is healthy control (HC). Then TP is MS correctly identified as MS, TN is HC correctly identified as HC, FN is MS falsely diagnosed as HC, FP is HC falsely diagnosed as MS. We define the sensitivity (Sen), specificity (Spc), and accuracy (Acc) on the basis of the realistic confusion matrix by following three formulae as:

(20) $$\mathrm{Sen}=\frac{\mathrm{TP}}{\mathrm{TP}+\mathrm{FN}}$$

(21) $$\mathrm{Spc}=\frac{\mathrm{TN}}{\mathrm{TN}+\mathrm{FP}}$$

(22) $$\mathrm{Acc}=\frac{\mathrm{TP}+\mathrm{TN}}{\mathrm{TP}+\mathrm{TN}+\mathrm{FP}+\mathrm{FN}}$$

**Figure 10:**
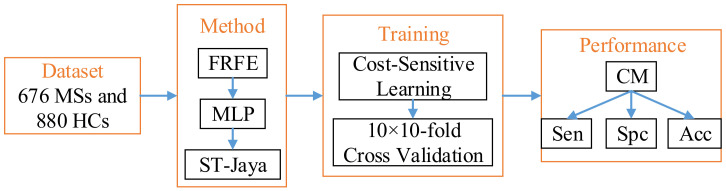
Flowchart of the proposed method.

We shall report the mean and standard deviation of above three measure indicators. Finally, the diagram of proposed method was drawn in Figure 10.

## 4. Experiments, Results, and Discussions

### 4.1. FRFE Map

**Figure 11:**
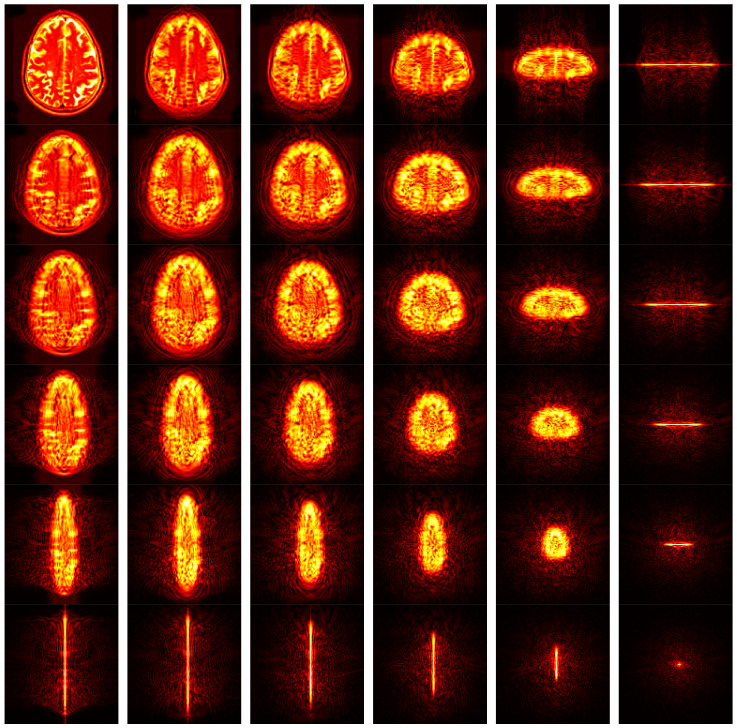
FRFT map of an MS brain image (hot colormap was added for better visual performance).

Figure 11 shows the FRFT map. The original brain image was shown in the top left corner, where the angle is (0, 0), corresponding to original spatial-domain image. From left to right, the first angle increases from 0 to 1 with increase of 0.2. From top to bottom, the second angle increases in similar way. The bottom right corner is associated with the FRFT map with angle of (1, 1).

Hence, the most prominent advantage of FRFT compared to other feature extraction methods, is it provides a continuous spectrum-like feature maps. Then, the entropy will further extract important information from those 36 spectrum maps. In all, we obtained a 36-element feature vector after this step.

Note that FRFE is still a hand-crafted feature, which has already been used for fine-grained classification, such as pathological brain detection [12], tea category identification [17], hearing loss identification [19], etc. Nevertheless, Riabchenko et al. [32] stated that they found superiority of the learned deep features to the engineered (viz., hand-crafted features), which suggests us to develop AI-based techniques to let the algorithm “learn” more efficient features than FRFE.

### 4.2. Statistical Analysis

The sensitivity, specificity, and accuracy results of 10 × 10-fold cross validation with cost-sensitive learning were shown in Table 2. The mean and standard deviation were listed in the caption. The final average result was Sen = 97.40 ± 0.60%, Spc = 97.39 ± 0.65%, Acc = 97.39 ± 0.59%. In the table R represents run, and F fold. We can observe the sensitivity is almost equivalent to the specificity, which indicates the success of our cost-sensitive learning technique.

### 4.3. ST-Jaya versus Plain Jaya

We compared the proposed ST-Jaya versus plain Jaya. We tested nine different settings of plain Jaya, in which two hyper-parameters need to be set beforehand (population size *S*, and number of hidden neurons NHN). The settings were listed in Table 3.

The Setting 2 achieved the best results among all nine settings. The statistical results of Setting 2 were listed in Table 4. It obtained a sensitivity of 97.03 ± 0.95%, a specificity of 97.05 ± 0.95%, and an accuracy of 97.04 ± 0.90%. Due to page limit, we only give the results other than the details of other eight settings, as shown in Table 5.

**Figure 12:**
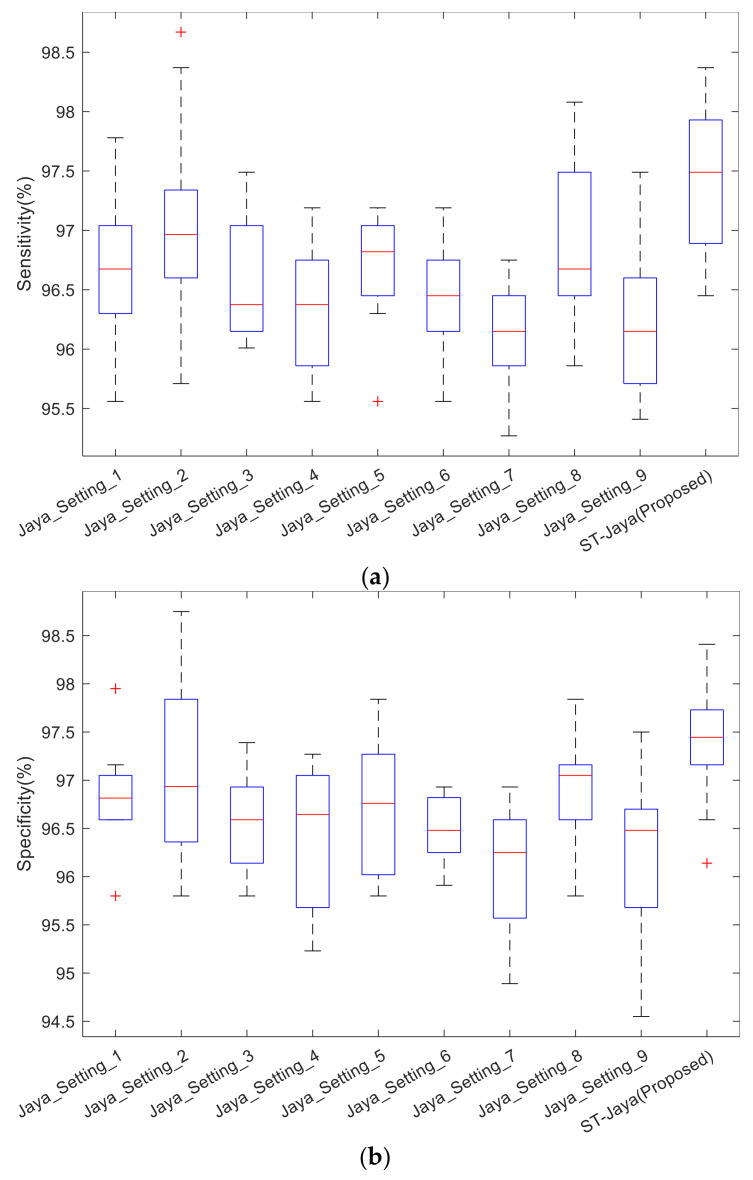

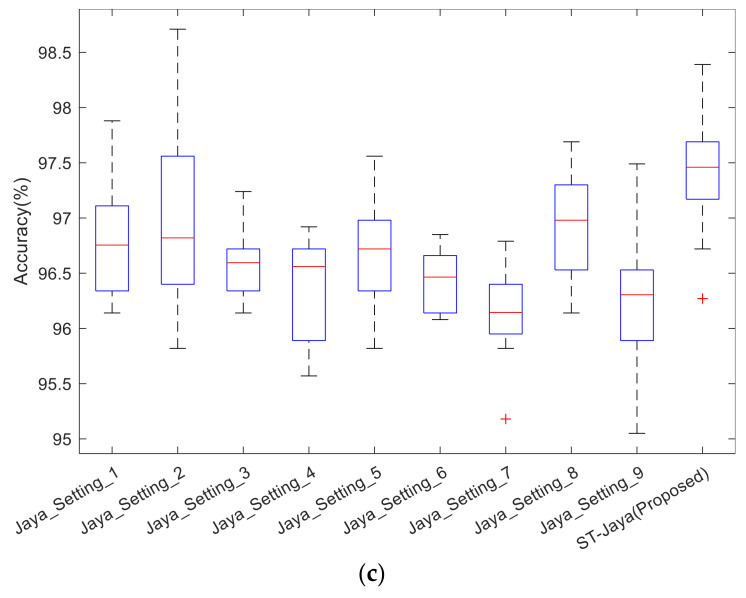
Boxplot of plain Jaya with proposed ST-Jaya: (**a**) sensitivity; (**b**) specificity; and (**c**) accuracy.

The boxplot was shown in Figure 12. Note that the central red line is median, not mean as shown in Table 5. The bottom and top edges indicate the 25th and 75th percentile. Outliers were marked individually using “+” mark. We observed from those boxplots that plain Jaya with any of nine different settings did not perform better than proposed ST-Jaya, which validated the superiority of proposed ST-Jaya to plain Jaya.

The success of ST-Jaya may attribute to the two factors: One is the self-adaptive strategy that avoid the users to set the value of population size, and the other is the three-segment-encoding strategy help the users to determine the number of hidden neuron automatically.

Except self-adaptive and three-segment-coding, there are other advanced strategies that may help bioinspired algorithms. Those strategies include adding chaotic [33] and/or fuzzy operators, opposition-based learning, manipulating topologic positions of population candidates, hybridization of other bioinspired algorithms. Those strategies may improve the convergence speed and the robustness of proposed ST-Jaya algorithm.

### 4.4. Comparison to Other Bioinspired Algorithms

**Figure 13:**
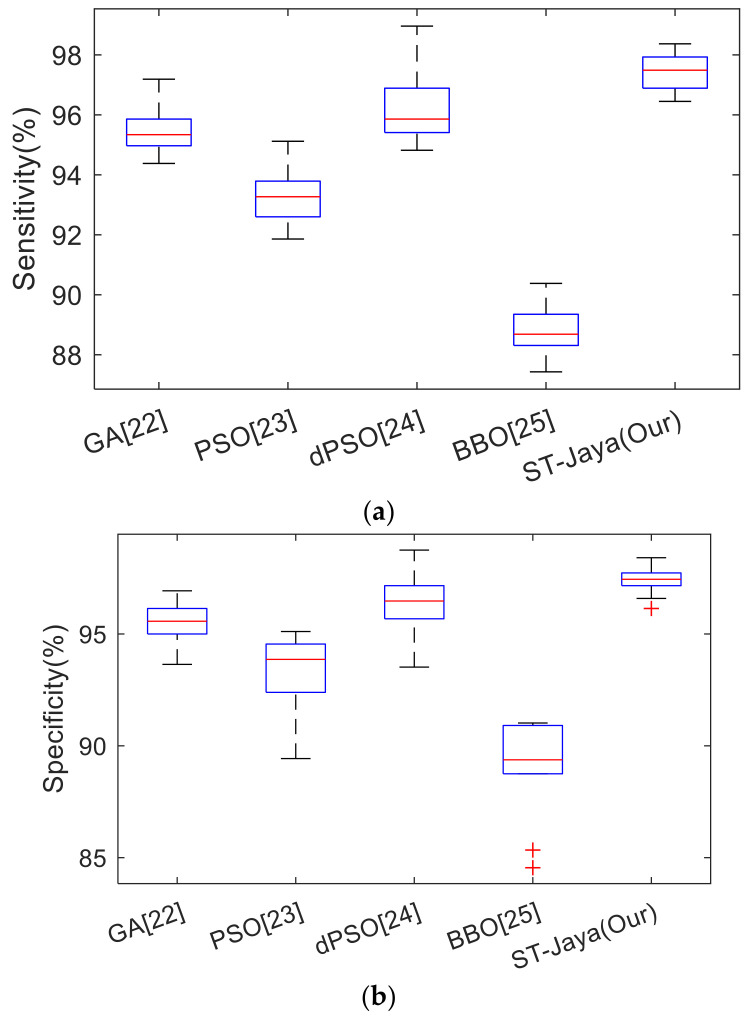

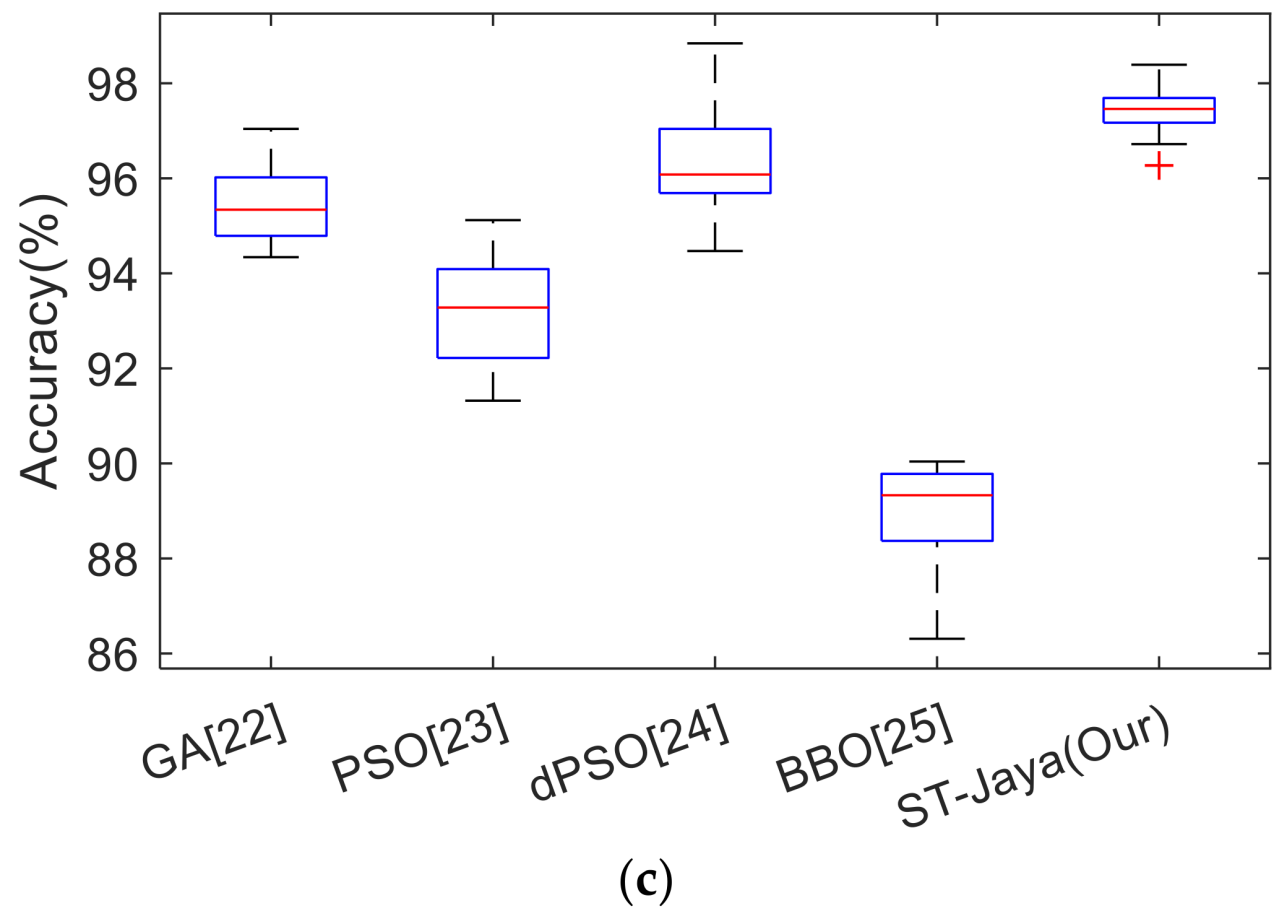
Boxplot of proposed ST-Jaya versus state-of-the-art bioinspired training methods. (**a**) Sensitivity; (**b**) Specificity; (**c**) Accuracy.

In this experiment, we shall compare the proposed ST-Jaya with state-of-the-art bio-inspired algorithms, including GA [22], PSO [23], dynamic PSO (dPSO) [24], and BBO [25]. Their full names can be found in Section 3.4. The optimal hyper-parameters of those four comparison basis methods were obtained by grid-search algorithm. The comparison results were listed in Table 6, and the corresponding boxplots were shown in Figure 13.

Next, we compared proposed ST-Jaya with those above four bioinspired methods: GA [22], PSO [23], dynamic PSO (dPSO) [24], and BBO [25] in terms of computation time. The 10 × 10-fold cross validation meant each algorithm ran 100 times. The average computation time of all methods are shown in Table 7.

The results in Table 7 indicated that GA [22] cost 25.54 ± 4.39 s, PSO [23] cost 16.08 ± 2.61 s, dPSO [24] cost 15.59 ± 3.17 s, BBO [25] cost 18.82 ± 3.80 s, while our proposed ST-Jaya cost the least time of only 13.77 ± 3.53 s. Hence, proposed ST-Jaya has a faster training speed than those four state-of-the-art approaches in training MLP.

Due to page limit, we only compared our proposed ST-Jaya with four bioinspired approaches. At present, there are numerous new bioinspired methods, such as gray wolf optimization, multi-verse optimizer, etc. Nevertheless, PSO is a classic and powerful swarm-intelligence method, and hence we included it as one of comparison algorithms. In our future studies, we shall extract the advantages of those latest algorithm, and try to improve the optimization performance of our proposed algorithm.

### 4.5. Comparison to Latest MS Identification Approaches

In this experiment, we compared the proposed MS identification method, FRFE + MLP + ST-Jaya, with state-of-the-art approaches, including GLCM-GLRL [8], MAMSM [9], RF [10], and HWT + LR [11]. Strict statistical analysis, i.e., the 10 × 10-fold cross validation was implemented. The mean and standard deviation values of sensitivities, specificities and accuracies of each algorithm, were reported in Table 8.

The results in Table 8 showed the worst algorithm was HWT + LR [11] with values less than 90%. All the other algorithms achieve performances greater than 90%. The second worst algorithm was MAMSM [9], which obtained a sensitivity of 93.24 ± 0.93%, a specificity of 93.15 ± 1.94%, and an accuracy of 93.19 ± 1.22%. The reason is the amplitude-modulation and frequency-modulation are originally designed for communication [34]. The algorithm with average performance was GLCM-GLRL [8], which combined gray-level cooccurrence matrix and gray-level run-length matrix. The second-best algorithm was the RF [10]. This method used random forest approach that is an ensemble learning method. The best algorithm was the proposed FRFE + MLP + ST-Jaya, and its success lie in the efficiency of FRFE map and the improvements of standard Jaya algorithm.

As a high-level view, our method shows handcrafted image features may give better performance than biomarkers in terms of classification performance. Besides, these handcrafted image features are accurate and reproducible. In the future, handcrafted features may be replaced by learned features by artificial intelligence techniques such as deep learning, which was a core technique in AlphaGo.

## 5. Conclusions and Future Direction

In this study, we proposed a method based on fractional Fourier entropy map, multilayer perceptron, and Jaya algorithm with two improvements. Our FRFE + MLP + ST-Jaya method achieved a promising result in identifying multiple sclerosis methods. The proposed parameter-free ST-Jaya has a faster MLP-training speed than other four state-of-the-art methods.

In the future, we may test other features that may help to extract more efficient MS-related characteristics, particularly wavelet-related [35] and entropy-related features [36]. Another research direction is to use deep learning technologies to this MS detection problem.

## Figures and Tables

**Table 1:** Analysis of our dataset.

Class	No. of Images	Cost
MS	676	1.30
HC	880	1

**Table 2:** Statistical results of proposed method (Sen = 97.40 ± 0.60, Spc = 97.39 ± 0.65, Acc = 97.39 ± 0.59).

**Sen**	**F1**	**F2**	**F3**	**F4**	**F5**	**F6**	**F7**	**F8**	**F9**	**F10**	**Total**
R1	95.52	95.59	98.51	98.51	97.06	94.12	97.06	97.01	97.06	94.12	96.45
R2	95.59	98.51	100.00	98.53	98.53	97.06	97.01	97.06	97.01	97.06	97.63
R3	97.06	94.12	95.59	97.06	98.53	100.00	100.00	98.53	98.51	100.00	97.93
R4	98.53	95.59	97.06	98.53	97.06	95.52	97.01	95.52	97.06	97.01	96.89
R5	95.52	97.06	98.53	95.59	100.00	98.53	97.01	97.01	98.51	97.06	97.49
R6	97.06	97.01	95.59	95.52	98.53	97.01	97.06	97.06	97.01	97.06	96.89
R7	98.53	95.59	97.01	95.52	97.06	98.51	97.06	97.06	97.01	95.59	96.89
R8	100.00	100.00	100.00	97.01	97.01	95.52	94.12	95.59	97.06	98.51	97.49
R9	95.52	100.00	97.06	98.53	98.51	98.53	98.53	100.00	98.51	98.53	98.37
R10	100.00	100.00	95.59	97.01	97.01	100.00	95.59	98.53	95.59	100.00	97.93
**Spc**	**F1**	**F2**	**F3**	**F4**	**F5**	**F6**	**F7**	**F8**	**F9**	**F10**	**Total**
R1	95.45	94.32	96.59	97.73	95.45	94.32	96.59	95.45	96.59	98.86	96.14
R2	98.86	96.59	97.73	96.59	97.73	96.59	100.00	97.73	97.73	97.73	97.73
R3	95.45	100.00	96.59	98.86	97.73	98.86	94.32	98.86	98.86	97.73	97.73
R4	97.73	97.73	97.73	96.59	98.86	96.59	96.59	97.73	97.73	97.73	97.50
R5	97.73	97.73	97.73	96.59	95.45	96.59	96.59	97.73	98.86	98.86	97.39
R6	97.73	97.73	96.59	97.73	96.59	97.73	98.86	96.59	98.86	95.45	97.39
R7	95.45	96.59	98.86	98.86	97.73	95.45	94.32	95.45	96.59	96.59	96.59
R8	95.45	98.86	97.73	97.73	97.73	97.73	97.73	98.86	97.73	98.86	97.84
R9	96.59	98.86	96.59	98.86	98.86	97.73	100.00	98.86	98.86	98.86	98.41
R10	96.59	97.73	97.73	95.45	96.59	96.59	96.59	98.86	97.73	97.73	97.16
**Acc**	**F1**	**F2**	**F3**	**F4**	**F5**	**F6**	**F7**	**F8**	**F9**	**F10**	**Total**
R1	95.48	94.87	97.42	98.06	96.15	94.23	96.79	96.13	96.79	96.79	96.27
R2	97.44	97.42	98.71	97.44	98.08	96.79	98.71	97.44	97.42	97.44	97.69
R3	96.15	97.44	96.15	98.08	98.08	99.35	96.77	98.72	98.71	98.71	97.81
R4	98.08	96.79	97.44	97.44	98.08	96.13	96.77	96.77	97.44	97.42	97.24
R5	96.77	97.44	98.08	96.15	97.44	97.44	96.77	97.42	98.71	98.08	97.43
R6	97.44	97.42	96.15	96.77	97.44	97.42	98.08	96.79	98.06	96.15	97.17
R7	96.79	96.15	98.06	97.42	97.44	96.77	95.51	96.15	96.77	96.15	96.72
R8	97.44	99.36	98.72	97.42	97.42	96.77	96.15	97.44	97.44	98.71	97.69
R9	96.13	99.35	96.79	98.72	98.71	98.08	99.36	99.36	98.71	98.72	98.39
R10	98.06	98.71	96.79	96.13	96.77	98.08	96.15	98.72	96.79	98.72	97.49

**Table 3:** Nine different configurations of plain Jaya.

Index	*S*	NHN
1	10	10
2	20	10
3	30	10
4	10	20
5	20	20
6	30	20
7	10	30
8	20	30
9	30	30

**Table 4:** Statistical results of the best Jaya with setting 2 (Sen = 97.03 ± 0.95, Spc =97.05 ± 0.95, Acc = 97.04 ± 0.90).

**Sen**	**F1**	**F2**	**F3**	**F4**	**F5**	**F6**	**F7**	**F8**	**F9**	**F10**	**Total**
R1	98.53	95.59	97.01	100.00	98.51	100.00	97.01	94.12	97.01	95.59	97.34
R2	98.51	95.59	95.59	100.00	97.01	98.51	94.12	95.59	97.01	98.53	97.04
R3	98.51	97.06	97.01	98.53	97.01	94.12	97.06	97.01	97.06	98.53	97.19
R4	94.12	95.52	95.52	97.06	95.52	94.03	97.06	95.59	95.59	97.06	95.71
R5	100.00	98.53	98.51	98.53	98.53	95.59	98.51	100.00	98.51	97.06	98.37
R6	94.12	98.53	97.01	95.59	98.51	98.53	100.00	97.06	92.54	97.01	96.89
R7	100.00	97.06	97.06	100.00	98.51	97.01	100.00	98.53	100.00	98.51	98.67
R8	94.12	97.01	97.01	95.59	94.12	97.06	95.52	94.03	98.53	95.59	95.86
R9	97.06	97.06	95.52	95.59	97.06	97.01	97.01	97.06	97.06	95.52	96.60
R10	98.53	98.53	94.03	94.12	98.51	94.03	94.12	98.51	97.06	98.53	96.60
**Spc**	**F1**	**F2**	**F3**	**F4**	**F5**	**F6**	**F7**	**F8**	**F9**	**F10**	**Total**
R1	95.45	97.73	97.73	96.59	97.73	94.32	97.73	100.00	97.73	96.59	97.16
R2	96.59	97.73	96.59	95.45	94.32	96.59	94.32	96.59	95.45	94.32	95.80
R3	97.73	96.59	96.59	98.86	98.86	96.59	100.00	97.73	98.86	96.59	97.84
R4	94.32	95.45	96.59	96.59	95.45	97.73	96.59	95.45	95.45	95.45	95.91
R5	100.00	95.45	100.00	98.86	98.86	98.86	95.45	98.86	95.45	98.86	98.07
R6	97.73	97.73	96.59	95.45	97.73	96.59	97.73	98.86	96.59	95.45	97.05
R7	98.86	100.00	98.86	97.73	98.86	96.59	98.86	98.86	100.00	98.86	98.75
R8	96.59	97.73	96.59	97.73	95.45	96.59	98.86	94.32	97.73	96.59	96.82
R9	98.86	95.45	98.86	95.45	96.59	98.86	95.45	94.32	97.73	95.45	96.70
R10	94.32	96.59	95.45	95.45	97.73	96.59	97.73	97.73	97.73	94.32	96.36
**Acc**	**F1**	**F2**	**F3**	**F4**	**F5**	**F6**	**F7**	**F8**	**F9**	**F10**	**Total**
R1	96.79	96.79	97.42	98.08	98.06	96.79	97.42	97.44	97.42	96.15	97.24
R2	97.42	96.79	96.15	97.44	95.48	97.42	94.23	96.15	96.13	96.15	96.34
R3	98.06	96.79	96.77	98.72	98.06	95.51	98.72	97.42	98.08	97.44	97.56
R4	94.23	95.48	96.13	96.79	95.48	96.13	96.79	95.51	95.51	96.15	95.82
R5	100.00	96.79	99.35	98.72	98.72	97.44	96.77	99.35	96.77	98.08	98.20
R6	96.15	98.08	96.77	95.51	98.06	97.44	98.72	98.08	94.84	96.13	96.98
R7	99.36	98.72	98.08	98.72	98.71	96.77	99.35	98.72	100.00	98.71	98.71
R8	95.51	97.42	96.77	96.79	94.87	96.79	97.42	94.19	98.08	96.15	96.40
R9	98.08	96.15	97.42	95.51	96.79	98.06	96.13	95.51	97.44	95.48	96.66
R10	96.15	97.44	94.84	94.87	98.06	95.48	96.15	98.06	97.44	96.15	96.47

**Table 5:** Comparison between plain Jaya and proposed ST-Jaya.

Training Algorithm	Sen	Spc	Acc
Jaya (Setting 1)	96.73 ± 0.73	96.84 ± 0.54	96.79 ± 0.53
Jaya (Setting 2)	97.03 ± 0.95	97.05 ± 0.95	97.04 ± 0.90
Jaya (Setting 3)	96.58 ± 0.52	96.60 ± 0.52	96.59 ± 0.34
Jaya (Setting 4)	96.32 ± 0.50	96.38 ± 0.79	96.35 ± 0.49
Jaya (Setting 5)	96.72 ± 0.50	96.72 ± 0.67	96.72 ± 0.49
Jaya (Setting 6)	96.43 ± 0.48	96.47 ± 0.36	96.45 ± 0.28
Jaya (Setting 7)	96.12 ± 0.47	96.11 ± 0.69	96.12 ± 0.44
Jaya (Setting 8)	96.88 ± 0.68	96.91 ± 0.60	96.90 ± 0.54
Jaya (Setting 9)	96.24 ± 0.66	96.24 ± 0.83	96.24 ± 0.65
ST-Jaya (Proposed)	97.40 ± 0.60	97.39 ± 0.65	97.39 ± 0.59

**Table 6:** Comparison between proposed ST-Jaya and other bio-inspired training algorithms.

Training Algorithm	Sen	Spc	Acc
GA [22]	86.79 ± 1.06	86.92 ± 1.05	86.86 ± 0.49
PSO [23]	95.38 ± 0.66	95.43 ± 0.97	95.41 ± 0.56
dPSO [24]	96.05 ± 0.91	96.01 ± 1.08	96.03 ± 0.88
BBO [25]	96.17 ± 0.62	96.22 ± 0.63	96.20 ± 0.53
ST-Jaya (Proposed)	97.40 ± 0.60	97.39 ± 0.65	97.39 ± 0.59

**Table 7:** Time analysis of MLP training methods of 100 runs.

Approach	Computation Time (Unit: s)
GA [22]	25.54 ± 4.39
PSO [23]	16.08 ± 2.61
dPSO [24]	15.59 ± 3.17
BBO [25]	18.82 ± 3.80
ST-Jaya (Proposed)	13.77 ± 3.53

**Table 8:** MS identification algorithm comparison.

MS Identification Method	Sen	Spc	Acc	Rank
GLCM-GLRL [8]	95.47 ± 0.81	95.48 ± 1.08	95.48 ± 0.80	3
MAMSM [9]	93.24 ± 0.93	93.15 ± 1.94	93.19 ± 1.22	4
RF [10]	96.23 ± 1.18	96.32 ± 1.48	96.28 ± 1.25	2
HWT + LR [11]	88.83 ± 0.90	88.95 ± 2.28	88.90 ± 1.20	5
FRFE + MLP + ST-Jaya (Proposed)	97.40 ± 0.60	97.39 ± 0.65	97.39 ± 0.59	1

## References

[1] Lublin F.D., Reingold S.C., Cohen J.A., Cutter G.R., Sørensen P.S., Thompson A.J., Wolinsky J.S., Balcer L.J., Banwell B., Barkhof F. (2014). Defining the clinical course of multiple sclerosis the 2013 revisions. Neurology.

[2] Costello F. (2016). Vision Disturbances in Multiple Sclerosis. Semin. Neurol..

[3] Pozzilli C. (2014). Overview of MS Spasticity. Eur. Neurol..

[4] Koutsis G., Kokotis P., Papagianni A.E., Evangelopoulos M.E., Kilidireas C., Karandreas N. (2016). A neurophysiological study of facial numbness in multiple sclerosis: Integration with clinical data and imaging findings. Mult. Scler. J..

[5] Sebastião E., Hubbard E.A., Klaren R.E., Pilutti L.A., Motl R.W. (2017). Fitness and its association with fatigue in persons with multiple sclerosis. Scand. J. Med. Sci. Sports.

[6] Zhou X.-X. (2016). Comparison of machine learning methods for stationary wavelet entropy-based multiple sclerosis detection: Decision tree, k-nearest neighbors, and support vector machine. Simulation.

[7] Zhan T.M., Chen Y. (2016). Multiple Sclerosis Detection Based on Biorthogonal Wavelet Transform, RBF Kernel Principal Component Analysis, and Logistic Regression. IEEE Access.

[8] Ghribi O., Sellami L., Slima M.B., Mhiri C., Dammak M., Hamida A.B. (2018). Multiple sclerosis exploration based on automatic MRI modalities segmentation approach with advanced volumetric evaluations for essential feature extraction. Biomed. Signal Process. Control.

[9] Murray V., Rodriguez P., Pattichis M.S. (2010). Multiscale AM-FM Demodulation and Image Reconstruction Methods with Improved Accuracy. IEEE Trans. Image Process..

[10] Nayak D.R., Dash R., Majhi B. (2016). Brain MR image classification using two-dimensional discrete wavelet transform and AdaBoost with random forests. Neurocomputing.

[11] Lopez M. (2017). Multiple Sclerosis Slice Identification by Haar Wavelet Transform and Logistic Regression. Adv. Eng. Res..

[12] Wang S., Zhang Y., Yang X., Sun P., Dong Z., Liu A., Yuan T.F. (2015). Pathological Brain Detection by a Novel Image Feature—Fractional Fourier Entropy. Entropy.

[13] MRI Lesion Segmentation in Multiple Sclerosis Database, in eHealth Laboratory, University of Cyprus. http://www.medinfo.cs.ucy.ac.cy/index.php/downloads/datasets.

[14] Loizou C.P., Pantziaris M., Seimenis I., Pattichis C.S. (2013). Brain MR image normalization in texture analysis of multiple sclerosis. J. Biomed. Gr. Comput..

[15] Dhal K.G., Quraishi M.I., Das S. (2017). An Improved Cuckoo Search based Optimal Ranged Brightness Preserved Histogram Equalization and Contrast Stretching Method. Int. J. Swarm Intell. Res..

[16] Lomax S., Vadera S. (2017). A Cost-Sensitive Decision Tree Learning Algorithm Based on a Multi-Armed Bandit Framework. Comput. J..

[17] Cattani C., Rao R. (2016). Tea Category Identification Using a Novel Fractional Fourier Entropy and Jaya Algorithm. Entropy.

[18] Sun Y. (2016). A Multilayer Perceptron Based Smart Pathological Brain Detection System by Fractional Fourier Entropy. J. Med. Syst..

[19] Li J. (2017). Texture Analysis Method Based on Fractional Fourier Entropy and Fitness-Scaling Adaptive Genetic Algorithm for Detecting Left-Sided and Right-Sided Sensorineural Hearing Loss. Fundam. Inform..

[20] Araujo R.D., Oliveira A.L.I., Meira S. (2017). A class of hybrid multilayer perceptrons for software development effort estimation problems. Expert Syst. Appl..

[21] Weiner B.C. (2017). Chronic obstructive pulmonary disease and Occam’s razor. Can. Med. Assoc. J..

[22] Zhao Y., Zhao H., Huo X., Yao Y. (2017). Angular Rate Sensing with GyroWheel Using Genetic Algorithm Optimized Neural Networks. Sensors.

[23] Kuok K.K., Harun S., Shamsuddin S.M. (2010). Particle swarm optimization feedforward neural network for modeling runoff. Int. J. Environ. Sci. Technol..

[24] Rakitianskaia A.S., Engelbrecht A.P. (2012). Training feedforward neural networks with dynamic particle swarm optimisation. Swarm Intell..

[25] Wu J. (2016). Fruit classification by biogeography-based optimization and feedforward neural network. Expert Syst..

[26] Rao R.V. (2016). Jaya: A simple and new optimization algorithm for solving constrained and unconstrained optimization problems. Int. J. Ind. Eng. Comput..

[27] Phillips P. (2018). Intelligent facial emotion recognition based on stationary wavelet entropy and Jaya algorithm. Neurocomputing.

[28] Ocłoń P., Cisek P., Rerak M., Taler D., Rao R.V., Vallati A., Pilarczyk M. (2018). Thermal performance optimization of the underground power cable system by using a modified Jaya algorithm. Int. J. Therm. Sci..

[29] Rao R.V., More K.C. (2017). Optimal design and analysis of mechanical draft cooling tower using improved Jaya algorithm. Int. J. Refrig..

[30] Chen X.-Q. (2016). Fractal dimension estimation for developing pathological brain detection system based on Minkowski-Bouligand method. IEEE Access.

[31] Pohjankukka J., Pahikkala T., Nevalainen P., Heikkonen J. (2017). Estimating the prediction performance of spatial models via spatial k-fold cross validation. Int. J. Geogr. Inf. Sci..

[32] Riabchenko E., Meissner K., Ahmad I., Iosifidis A., Tirronen V., Gabbouj M., Kiranyaz S. Learned vs. Engineered Features for Fine-Grained Classification of Aquatic Macroinvertebrates. Proceedings of the 23rd International Conference on Pattern Recognition (ICPR).

[33] Hou X.-X. (2017). Alcoholism detection by medical robots based on Hu moment invariants and predator-prey adaptive-inertia chaotic particle swarm optimization. Comput. Electr. Eng..

[34] Kindness S.J., Jessop D.S., Wei B., Wallis R., Kamboj V.S., Xiao L., Ren Y., Braeuninger-Weimer P., Aria A.I., Hofmann S. (2017). External amplitude and frequency modulation of a terahertz quantum cascade laser using metamaterial/graphene devices. Sci. Rep..

[35] Yang M. (2016). Dual-Tree Complex Wavelet Transform and Twin Support Vector Machine for Pathological Brain Detection. Appl. Sci..

[36] Yang J. (2015). Preclinical diagnosis of magnetic resonance (MR) brain images via discrete wavelet packet transform with Tsallis entropy and generalized eigenvalue proximal support vector machine (GEPSVM). Entropy.

